# Integrated Metabolomics and Transcriptomics Suggest the Global Metabolic Response to 2-Aminoacrylate Stress in *Salmonella enterica*

**DOI:** 10.3390/metabo10010012

**Published:** 2019-12-24

**Authors:** Andrew J. Borchert, Jacquelyn M. Walejko, Adrien Le Guennec, Dustin C. Ernst, Arthur S. Edison, Diana M. Downs

**Affiliations:** 1 Department of Microbiology, University of Georgia, Athens, GA 30602, USA; aborch@uga.edu (A.J.B.); dernst@ucsd.edu (D.C.E.); 2 Department of Biochemistry & Molecular Biology, Complex Carbohydrate Research Center, University of Georgia, Athens, GA 30602, USA; jacquelyn.walejko@duke.edu (J.M.W.); adrien.le_guennec@kcl.ac.uk (A.L.G.); 3 Departments of Genetics and Biochemistry & Molecular Biology, Institute of Bioinformatics, Complex Carbohydrate Research Center, University of Georgia, Athens, GA 30602, USA; aedison@uga.edu

**Keywords:** 2-aminoacrylate stress, RidA, pyridoxal 5′-phosphate, metabolic networks, metabolomics, ^1^H NMR

## Abstract

In *Salmonella enterica*, 2-aminoacrylate (2AA) is a reactive enamine intermediate generated during a number of biochemical reactions. When the 2-iminobutanoate/2-iminopropanoate deaminase (RidA; EC: 3.5.99.10) is eliminated, 2AA accumulates and inhibits the activity of multiple pyridoxal 5’-phosphate(PLP)-dependent enzymes. In this study, untargeted proton nuclear magnetic resonance (^1^H NMR) metabolomics and transcriptomics data were used to uncover the global metabolic response of *S. enterica* to the accumulation of 2AA. The data showed that elimination of RidA perturbed folate and branched chain amino acid metabolism. Many of the resulting perturbations were consistent with the known effect of 2AA stress, while other results suggested additional potential enzyme targets of 2AA-dependent damage. The majority of transcriptional and metabolic changes appeared to be the consequence of downstream effects on the metabolic network, since they were not directly attributable to a PLP-dependent enzyme. In total, the results highlighted the complexity of changes stemming from multiple perturbations of the metabolic network, and suggested hypotheses that will be valuable in future studies of the RidA paradigm of endogenous 2AA stress.

## 1. Introduction

The metabolic network of an organism consists of a complex system of biochemical reactions that together result in the behavioral characteristics of the organism. In general, the reactions that make up the metabolic network, and their integration, are governed by chemical and physical constraints placed on the cell by its environment. Microbes in particular face rapidly changing environments and their metabolic networks must be structured to absorb and/or respond to internal and external insults [1,2,3]. Technological advances continue to expand and provide deeper and more detailed global snapshots of features in a cell or population. As these large datasets accumulate, the focus turns to analyzing these data as a means to glean insights about fundamental processes in biological systems. To that end, untargeted metabolomics and comparative transcriptomics data are increasingly being used as the basis for building metabolic models that describe the physiological state of a cell [4,5,6]. Examples of this approach can be found in disciplines ranging from mammalian genetic disorders and cancer biology, to antibiotic modes of action and pathogen–host interaction [7,8,9,10]. While these analyses can contribute to defining the response to various cellular perturbations [11,12], they are most significant when confirmed by rigorous genetic, biochemical, and molecular biological experimentation. In theory, the combination of global discovery approaches with focused reductionist analyses provides an optimal approach to generate insights into the fundamentals of the metabolic network structure and cellular strategies [13,14].

**Figure 1:**
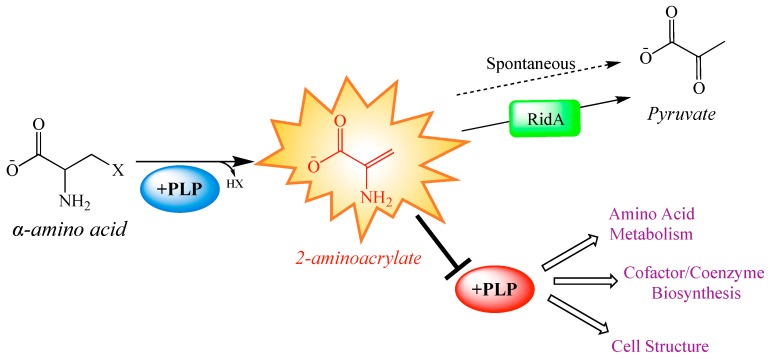
Paradigm 2-aminoacrylate stress in *Salmonella enterica.* Some pyridoxal 5′-phosphate (PLP)-dependent enzymes catalyze the β-elimination of 3-carbon α-amino acids, generating and releasing a reactive enamine product, 2-aminoacrylate (2AA). 2AA can be hydrolyzed to pyruvate by a non-enzymatic, or RidA catalyzed process. In the absence of RidA, 2AA can accumulate endogenously, where it can attack the active site of various PLP-dependent enzymes and inactive them with covalent modification. Generally, PLP-dependent enzymes play biochemical roles related to amino acid metabolism, cofactor and coenzyme production, and cell structure synthesis. Consequently, many of the pathways making up these broad physiological processes are perturbed by the cellular accumulation of 2AA [21,22,23,25].

An important use of global strategies is to expand the scope of a model that has been based on data from biochemical genetic approaches, which often define detail but can lack validation on a global scale. The RidA paradigm of enamine stress in *Salmonella enterica* defines a metabolic model with clearly defined local effects that result in predictable global consequences. Members of the broadly conserved RidA protein family are responsible for the hydrolysis of enamine/imine species, which are generated as intermediates in a variety of biochemical reactions [15,16,17,18,19,20] (Figure 1). In the absence of RidA, the enamine 2-aminoacrylate (2AA), a well-characterized inhibitor of multiple pyridoxal 5′-phosphate (PLP)-dependent enzymes, accumulates (Figure 1). Accumulated 2AA inhibits serine hydroxymethyltransferase (GlyA, EC: 2.1.2.1), aspartate aminotransferase (AspC; EC: 2.6.1.1), alanine racemase (Alr and DadX; EC: 5.1.1.1), and branched-chain amino acid aminotransferase (IlvE; EC: 2.6.1.42) [21,22,23,24], reducing their total cellular activity by 20–60%.

Overall, *S. enterica* has over 40 different PLP-dependent enzymes with functions in amino acid metabolism, cofactor/coenzyme biosynthesis, and the synthesis of cell structure components [26,27]. While a handful of specific 2AA targets have been defined, less is known about the extent of 2AA-dependent damage or how such damage, (i.e., enzyme inhibition) impacts the metabolic network. Further, 2AA stress is expected to elicit a broad shift in the metabolome, observed as the cumulative response to damaging a subset of PLP-dependent enzymes. A previous comparative transcriptomics study highlighted transcriptional changes in a strain lacking *ridA*, and led to the identification of an uncharacterized role for RidA in motility [28]. A majority of the most significant transcriptional changes (fold-change > 2) had no direct connection to a PLP-dependent enzyme, suggesting they were a downstream consequence of 2AA damage.

This study was initiated to further our understanding of the global metabolic response of *S. enterica* to 2AA stress. Specifically, untargeted NMR metabolomic data were sought to complement existing transcriptomics data. Our understanding of the RidA system at a physiological, genetic, and biochemical level provided a critical basis to guide the interpretation of the global data sets.

## 2. Results and Discussion

### 2.1. Metabolite Levels Are Altered in an S. enterica RidA Mutant

Comprehensive ^1^H NMR analysis was performed on the cellular contents of an isogenic pair of *S. enterica* LT2 strains that differed only at the *ridA* locus (DM9404 and DM3480) after growth to stationary phase in minimal glucose medium. These data were viewed through a biological lens, specifically considering the role PLP-enzyme-dependent enzymes have in the *ridA* paradigm [29,30], to extract insights into the effect of 2AA stress on the metabolic network that goes beyond information gleaned from past genetic, biochemical, and transcriptomic analyses.

In total, 37 metabolites could be structurally identified from the ^1^H-NMR spectral data (Table 1 and Table 2). Table 1 contains data from the 23 metabolites with significantly altered concentrations between wild-type and *ridA* strains, using a false discovery rate (FDR) adjusted *p* value < 0.05. Table 2 shows the 14 metabolites detected in all samples that did not meet the statistical threshold for a significant difference between strains. The data in Table 1 emphasize that the metabolic environment in a cell is impacted by the status of *ridA*. The relevant metabolites represented diverse areas of the metabolic network and did not suggest a simple model for specific site(s) of 2AA-dependent perturbation. A majority of the metabolites (10/23) were related to, or the product of, amino acid biosynthetic pathways and a significant number (5/23) were components nucleotide metabolism. The former was expected based on the prevalence of PLP-dependent enzymes (targets of 2AA damage) in amino acid metabolism [22,23,24,25,31], while a direct role of PLP-dependent enzymes in nucleotide metabolism was not obvious. The 37 metabolites that could be confidently identified represented a small sample from all metabolites produced by *S. enterica* and thus limited the conclusions that could be made about the global metabolic state of *S. enterica ridA* mutants.

### 2.2. Transcriptome Analyses of RidA Mutant Complements Metabolomics Data

A transcriptomic dataset obtained with the same wild-type and *ridA* mutant strains [28] was considered in combination with the metabolomics data to build a more complete picture of the metabolic changes that reflect the cellular response to 2AA accumulation. All gene expression data discussed were extracted from Borchert and Downs, 2017 [28] and is accessible at the National Center for Biotechnology Information Gene Expression Omnibus (NCBI GEO) (GSE103146; http://www.ncbi.nlm.nih.gov/geo/query/acc.cgi?acc=GSE103146). As reported, the transcription of 186 genes in a *S. enterica ridA* mutant were significantly (FDR < 0.05) elevated and the transcription of 227 genes was significantly decreased when compared to the wild-type parental strain [28]. Of the 413 genes that were differentially expressed, 113 had a greater than 2-fold difference in expression between *ridA* and WT. These data emphasized that the cellular response to 2AA stress generated transcriptional changes of modest magnitude. Over-representation analysis was performed on all differentially expressed genes, regardless of fold-change intensity, to determine pathways that showed significant enrichment for the differentially expressed genes in a *ridA* mutant (Table 3) [32,33]. These analyses confirmed that gene networks involved in flagellar biosynthesis, chemotaxis, and epithelial cell invasion were significantly down-regulated in a *ridA* mutant, observations that previously led to the characterization of a motility defect in *ridA* mutants [28]. Genes encoding ribosomal proteins were also significantly down-regulated (Table 3). Since ribosome synthesis is tightly regulated in accordance with growth rate and amino acid availability, [34,35,36] this change was assumed to reflect the general cell status of *ridA* mutants, i.e., perturbed amino acid metabolism and reduced growth rate [25].

Over-representation analysis showed the KEGG pathways for thiamine metabolism (stm00730), biotin metabolism (stm00780), amino acid biosynthesis (stm01230), one-carbon metabolism (stm00670), ABC transporters (stm02010), and glycine, serine and threonine metabolism (stm00260) were all significantly over-represented as up-regulated in a *ridA* mutant (Table 3). Since many of these KEGG pathways contain PLP-dependent enzymes, they were the focus of hypotheses to define the metabolic perturbations resulting from 2AA accumulation. In cells that lack RidA, accumulated 2AA targets PLP-dependent enzymes, and precedent with IlvE, GlyA, and Alr [21,22,23] suggests the activity of their population is decreased by 20–60%. The decreased activity is expected to collectively dampen flux in multiple metabolic nodes and cause metabolite imbalance(s). A subset of these imbalances would be detectable by an untargeted metabolomics approach. Such metabolite imbalances have the potential to trigger transcriptional regulatory responses, such as those identified in the transcriptome data. 

### 2.3. Folate Metabolism Is Perturbed in a RidA Mutant

**Figure 2:**
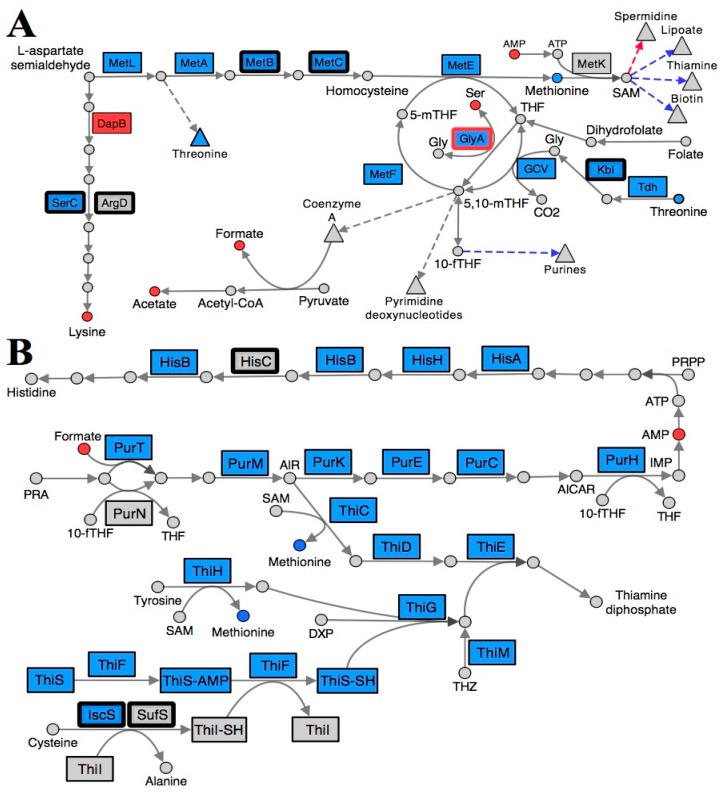
Folate and S-adenosylmethionine pathways are reorganized in a *S. enterica ridA* mutant. Metabolites (circles) and gene products (rectangles) involved in (**A**) lysine, methionine, and folate biosynthesis and (**B**) purine, histidine, and thiamine biosynthesis are depicted, with each arrow representing a biochemical step in the pathway. Blue shading denotes a significantly (FDR < 0.05) increased abundance of the respective metabolite/transcript in *ridA* cells, while red shading indicates a significantly decreased abundance in *ridA* cells. Grey gene products showed no significant difference between wild-type and *ridA* cells, while grey metabolites were either not identified or not significantly altered. A bold border around a gene product designates enzymes that use PLP as a cofactor. GlyA is a known target for 2AA damage and is shown with a red border. Dashed arrows and triangles indicated general pathways which utilize the metabolite at the base of the arrow, while blue and red colored arrows denote genes involved in biosynthesis of that compound up- or down-regulated, respectively.

**Figure 3:**
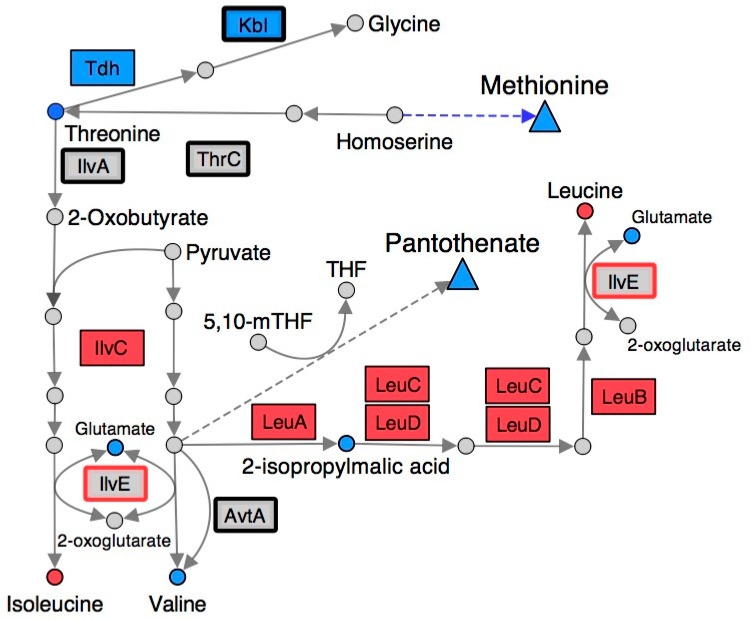
Gene expression and metabolite abundance in branched chain amino acid metabolism are altered in a *S. enterica ridA* mutant. Metabolites and gene products involved in branched-chain amino acid biosynthesis are shown using circles and rectangles, respectively. Blue shading represents significantly (FDR < 0.05) increased abundance of the respective metabolite/transcript in *ridA* cells, while red shading indicates significantly decreased abundance in *ridA* cells. Grey gene products showed no significant difference between wild-type and *ridA* cells, while grey metabolites were either not identified or not significantly altered. A bold border around a gene product denotes enzymes that use PLP as a cofactor. IlvE (red border) is a known target for 2AA damage. Dashed arrows and triangles indicated general pathways that utilize the metabolite at the base of the arrow.

Collectively, the transcriptional and metabolic shifts detected in a *ridA* mutant are consistent with a perturbation in folate metabolism. Tetrahydrofolate (THF) and its derivatives are essential cofactors used to facilitate one-carbon unit transfers in many important pathways [37]. The transcription of multiple genes involved in 5,10-methylenetetrahydrofolate (5,10-mTHF) biosynthesis (*glyA*, *gcvTHP*, *tdh*, *kbl*, *pabB*, and *folE*) were increased in a *ridA* mutant, as compared to wild-type (Table 3, Figure 2A). This expression profile indicates a response in cells that have been compromised in THF pools. Extensive biochemical genetic analyses support this finding, showing that accumulated 2AA from an *S. enterica ridA* mutant inhibits GlyA and reduces both glycine and 5,10-mTHF levels [22,25]. Interestingly, the expression of *glyA* and *gcvTHP* are induced in a *ridA* mutant, a response which may serve a role in counteracting the consequence of 2AA-dependent GlyA damage. The genes encoding threonine dehydrogenase (Tdh; EC: 1.1.1.103) and 2-amino-3-ketobutyrate CoA ligase (Kbl; EC: 2.3.1.29), which are involved in the conversion of threonine to glycine, are also induced in a *ridA* mutant. Induction of these genes could lead to the rerouting of threonine for glycine production, ultimately generating additional 5,10-mTHF via the glycine cleavage complex (GCV) (Figure 2A and Figure 3). Transcription of *pabB* (1.45-fold) and *folE* (3.21-fold), is also elevated in a *ridA* mutant. Expression of these genes, which are involved in 7,8-dihydrofolate production, could increase the biosynthesis of THF for use by GlyA and the GVC complex during 5,10-mTHF production. The putative 5,10-mTHF limitation in a *ridA* mutant would explain the increased expression of multiple pathways that are dependent upon folate metabolism (methionine, purine, histidine, thiamine, biotin, and lipoate biosynthesis) (Figure 2). 

Further, the transcriptional repressor, MetJ, binds S-adenosylmethionine (SAM) and represses expression of a number of genes whose expression is increased in a *ridA* mutant (*metA*, *metBL*, *metC*, *metE*, *metF, metMIQ*, *metR,* and *folE*) [28,38]. Increased expression of these MetJ/SAM regulated genes suggests that SAM is limiting in the *ridA* background. In fact, SAM biosynthesis is reliant on both 5-methyltetrahydrofolate (5-mTHF) and purines (ATP), making this conclusion consistent with the data and interpretations above. An expected consequence of SAM limitation is the decreased production of products of pathways that rely upon SAM as a cofactor. Consistently, expression of genes involved in the SAM-dependent pathways of biotin (*bioA*, *bioBFCD*, and *bisC*), lipoate (*lipA*), and thiamine (*thiCEFSGH* and *thiMD*) biosynthesis were induced, consistent with a limitation for these critical metabolites in a *ridA* mutant (Figure 2B).

Finally, ^1^H NMR metabolomic analysis showed that a *ridA* mutant had lower endogenous levels of both formate and acetate (Table 1). During mixed acid fermentation, pyruvate-formate lyase (PflB: EC: 2.3.1.54), uses Coenzyme A (CoA) and pyruvate as substrates during the production of formate and acetyl-CoA. Acetyl-CoA is further processed to acetate. Importantly, previous characterization of an *S. enterica ridA* mutant has revealed that 2AA-dependent damage of GlyA, and the resulting defect in 1-carbon unit production leads to a bottleneck in CoA biosynthesis [22]. Therefore, the reduced formate and acetate content in a *ridA* mutant could be explained by a limitation of CoA in a *ridA* mutant.

### 2.4. Branched-Chain Amino Acid (BCAA) Metabolism Is Altered in a RidA Mutant

Some of the most significant changes, by magnitude, in a *ridA* mutant involved metabolites related to branched-chain amino acid biosynthesis (Figure 3). Metabolomic analysis with ^1^H NMR showed that isoleucine and leucine content were decreased in a *ridA* mutant, while valine, 2-isopropylmalic acid, and threonine were increased (Table 1). IlvE is a target of 2AA damage and the resulting decrease in activity could contribute to the reduced levels of leucine and isoleucine [21]. Down-regulation of the *leu* operon and *ilvC* could also play a role in reducing leucine and isoleucine content in the *ridA* mutant background (Figure 3). Pyruvate accumulates during exponential growth of *ridA* mutants as a result of CoA limitation [22], and since pyruvate accumulation can increase flux toward L-valine biosynthesis [39,40], this could lead to the increased valine and 2-isopropylmalic acid levels observed in a *ridA* mutant (Table 1). As described above, the final steps of leucine and isoleucine biosynthesis involve a known target for 2AA damage (IlvE). It is possible that constriction of leucine and isoleucine generation, owing to damage of IlvE by 2AA, causes the accumulation of their precursors, 2-isopropylmalate and threonine, respectively (Figure 3).

### 2.5. Many Metabolic Perturbations Consistent with Transaminase Damage by 2AA

The ^1^H NMR metabolomics data indicated there was a decrease in lysine, serine, and phenylalanine, and an increase in glutamate in a *ridA* mutant. Synthesis of lysine, serine, and phenylalanine all rely upon the activity of PLP-dependent transaminases, which use glutamate as an amino group donor [41,42,43]. Thus, these changes could reflect damage to one or more of these transaminases by 2AA. For example, IlvE, AspC, and aromatic amino acid aminotransferase (TyrB; EC: 2.6.1.57) facilitate the synthesis of phenylalanine from phenylpyruvate [42]. Damage of IlvE [21] and AspC [24] by 2AA may explain the phenylalanine decrease in a *ridA* background. Similarly, phosphoserine transaminase (SerC, EC: 2.6.1.52) facilitates the conversion of 3-phosphooxypyruvate to O-phospho-L-serine during serine biosynthesis, while SerC and N-succinyldiaminopimelate aminotransferase (ArgD, EC: 2.6.1.17) catalyze N-succinyldiaminopimelate production from N-succinyl-L-amino-6-ketopimelate during lysine biosynthesis (Figure 3) [41,43]. The susceptibility of SerC to 2AA damage has not been confirmed, but reduced SerC activity would be expected to decrease both serine and lysine levels.

## 3. Materials and Methods

### 3.1. Bacterial Strains, Chemicals and Media

Strains used in this work are derivatives of *Salmonella enterica* subsp. *enterica* serovar Typhimurium str. LT2. The *ridA* null mutant (DM3480) used in this study contains a MudJ1734 transposon insertion [44] disrupting the *ridA* locus (*ridA3*::MudJ1734) [45]. Strain DM9404 is isogenic to DM3480 and has a wild-type *ridA* locus. Minimal medium was no-carbon E medium (NCE) containing 1 mM MgSO4 [46], trace metals [47], and 11 mM D-glucose. Difco nutrient broth (NB) (8 g/L) supplemented with 5 g/L NaCl was used as rich medium. All chemicals were purchased from the Sigma-Aldrich Chemical Company (St. Louis, MO, USA).

### 3.2. Metabolomics Cell Preparation

Ten biologically independent cultures each of wild-type (DM9404) and *ridA* mutant (DM3480) strains were grown aerobically overnight in NB medium at 37 °C and used to inoculate (1% inoculum) 250 mL minimal glucose medium in 500 mL non-baffled flasks. Flasks were randomly arranged in an Innova^®^ 44 incubator and cultures were allowed to grow to stationary phase (16 h) shaking at 180 RPM and 37 °C. Cultures were cooled on ice for 5 min and then harvested by centrifugation at 7000× *g* for 10 min at 4 °C. The supernatant was decanted, pellets were resuspended in 10 mL ddH_2_O, and transferred to sterile 15 mL conical tubes in which they were pelleted at 7000× *g* 10 min at 4 °C. Final supernatant was decanted before pellets were frozen in liquid nitrogen and stored at −80 °C prior to cell extractions.

### 3.3. Metabolite Extraction

Each frozen bacterial pellet (~1 g) was thawed on ice and homogenized 3 times. For homogenization, 2.6 g of 0.1 mm zirconium beads and 6 mL 4 °C MeOH/H_2_O (80/20) solvent was added and samples were agitated 7 times (210 s total) at 1600 rpm in a FastPrep 96 (MPBIO). Samples were then centrifuged at 416× *g* and 4 °C for 16 min and supernatant was transferred to a new 15 mL conical tube. Homogenization was carried out a second and third time using 4 mL MeOH/H_2_O with 4 homogenization cycles (150 s) and 2 mL MeOH/H_2_O with 3 homogenization cycles (150 s), respectively. Pooled supernatants from each sample were concentrated to dryness using a CentriVap Benchtop Vacuum Concentrator (Labconco). The extracts were reconstituted in 600 μL of deuterated 100 mM sodium phosphate buffer (pH 7.4) containing 1 mM of the internal standard DSS (d6 4,4-dimethyl-4-silapentane-1-sulfonic acid) and vortex mixed 2 min. Each sample was transferred into 5 mm SampleJet NMR tubes for NMR analysis.

### 3.4. Acquisition and Processing of NMR Spectral Data

#### 3.4.1. 1D ^1^H

The 1D proton spectra were collected using the pulse sequence ‘noesypr1d’ from the Bruker library. A mixing time of 5 ms was used, and during the acquisition, 65,536 complex datapoints were collected for the FID using 128 scans, with four additional dummy scans for equilibration and 4 s between scans. The spectral width was 20 ppm. For processing, 32,768 points were used for the spectrum, using an exponential window function of 0.3 Hz before the Fourier transform (FT). After FT and phase correction, a polynomial baseline correction of order 3 was used, and the frequency calibrated to the DSS peak (0.00 ppm).

#### 3.4.2. J-RES

The 2D J-RES spectra were collected using the pulse sequence ‘jresgpprqf’ from the Bruker library. 32,768 points were used for the FID in the direct dimension and 64 points for the indirect dimension, with a spectral width of 20 ppm for the direct dimension and 50 Hz for the indirect dimension. Eight scans were acquired for each t_1_ increment, along with four dummy scans for the equilibrium and 2 s between scans. For processing, 32,768 points were used for the direct dimension of the spectrum and 512 points for the indirect dimension. An unshifted sine window function was also used in each dimension, along with backward linear prediction of 64 points in the indirect dimension to increase the sensitivity as well as the resolution [48]. A polynomial baseline correction of order 3 in the direct dimension and order 5 in the indirect dimension was applied after the FT in both dimensions. Spectra were processed using in-house MATLAB scripts (https://github.com/artedison/Edison_Lab_Shared_Metabolomics_UGA) and all raw and processed metabolomics data are available on the Metabolomics workbench (http://www.metabolomicsworkbench.org/).

### 3.5. Compound Identification/Database Matching

Two-dimensional ^1^H-^13^C heteronuclear single quantum correlation (HSQC) and ^1^H-^13^C HSQC–TOCSY (HSQC–total correlation spectroscopy) experiments were used to aid in annotation of 2D J-RES metabolites and were not used for statistical analysis. A total of 37 metabolites were identified in bacterial extractions using COLMARm [49], comprehensive metabolite identification strategy using multiple two-dimensional NMR spectra of a complex mixture implemented in the COLMARm Web Server and assigned a confidence level ranging from 1 to 5, as previously described [50]. In short: (1) putatively characterized compound, (2) matched reported 1D spectra, (3) matched reported HSQC spectra, (4) matched reported HSQC and HSQC-TOCSY spectra, and (5) validated by spiking putative compound into sample. A list of these assignments can be found in Supplementary Table S1. In addition to the database matching, using 2D NMR data, we identified nicotinate mononucleotide, which was not in a spectral database. We verified this identification with a spiking experiment (Supplementary Figure S1).

### 3.6. Quantification of Metabolomics Data

Two-dimensional (2D) J-RES spectra were used for quantification and annotation of metabolites. A representative 2D J-RES spectra, with significantly altered metabolites annotated, is provided in Figure S2. The binned feature used for integration of each metabolite included in the statistical analysis are listed in Table S1. Univariate statistics were performed on metabolites identified using 2D J-RES data after PQN-normalization. A student’s t-test with an FDR correction was used to determine metabolites significantly altered in bacterial extracts from wild-type (n = 9) and *ridA* mutants (n = 10; FDR-corrected *p* value < 0.05).

### 3.7. Pathway Analysis

Kyoto Encyclopedia of Genes and Genomes (KEGG) pathways that were induced or repressed in *S. enterica ridA* mutants were determined using the Database for Annotation, Visualization and Integrated Discovery (DAVID) pathway enrichment analysis tool [32,33]. Input was a list of the locus tags (STM numbers) from the significantly (FDR < 0.05) differentially regulated genes separated into “up-regulated” and “repressed” subsets. Of the 413 locus tags used as input, 228 (55.2%) could be placed into a KEGG pathway. The output for up-regulated and down-regulated genes and which KEGG pathway they mapped to is provided in Supplementary File 1 (Supplementary Tables S2 and S3, respectively). These tables contain values representing the total gene count of pathway, hit counts, over-representation analyses (ORA) of raw *p* values, gene identities for hits, and ORA Benjamini-Hochberg FDR values [51]. Significantly over-represented pathways were defined as pathways with an FDR < 0.1. Figure 2 and Figure 3 were generated using Cytoscape 3.7.1 software [52].

## 4. Conclusions

The current study used comparative transcriptomics and untargeted ^1^H NMR metabolomics analysis to uncover global metabolic consequences of removing RidA. Importantly, all *S. enterica ridA* mutant-associated phenotypes to date are not only eliminated by expression of wild-type *ridA in trans*, but exist as the consequence enamine species (2-aminoacrylate) accumulation [15,17,22,23,25,28,31,45,53,54,55]. The transcriptional changes seen for a *ridA* mutant permeated multiple KEGG pathways, including amino acid, coenzyme, and folate metabolism. Complementing this finding, ^1^H NMR revealed multiple metabolic changes that were involved in amino acid, mixed acid fermentation, and nucleic acid metabolism. Some of the patterns from the transcriptomics and metabolomics data were explained by extrapolating known consequences from 2AA stress. For instance, induced expression of folate-related genes can be tied to damage of GlyA, and decreased isoleucine and leucine content with damage to IlvE. Indeed, many of the shifts in threonine, serine, lysine, and phenylalanine were consistent with the 2AA-dependent damage of PLP-dependent enzymes involved in the metabolism of these metabolites.

Significantly, numerous other metabolic and transcriptional changes in a *ridA* mutant could not easily be attributed to the inhibition of one or more PLP-dependent enzyme(s), the known targets of 2AA. These changes are expected to be the consequence of downstream effects on the metabolic network. Importantly, while they are difficult to tie to a specific 2AA target, these effects often prove important for the physiological state of the organism and cause visible phenotypes. This study took advantage of a mechanistically understood but complex paradigm of metabolic stress. The accumulation of 2AA causes a cellular stress that is multipronged, yet subtle, since the activity of multiple enzymes is reduced by 20–60%. The data herein showed that defining changes to the metabolic network created by multiple small perturbations to the system is not straightforward with the tools currently available. The RidA system can provide a valuable template for the continued refinement of global approaches to understand metabolic network structure, since it is understood at a biochemical level and can be manipulated genetically to test hypotheses that arise.

## Figures and Tables

**Table 1:** Significantly altered (FDR < 0.05) metabolites in *S. enterica ridA* background.

^1^ Compound	^2^ Mean (SD)		^3^ Fold Change	*p* Value	FDR Value
	Wild-Type	*ridA*			
Valine	1.23 (0.13)	2.71 (0.13)	2.20	8.47 × 10^−14^	3.56× 10^−12^
Methionine	0.08 (0.01)	0.17 (0.01)	2.20	4.43 × 10^−12^	9.31× 10^−11^
GSSG	0.54 (0.20)	2.02 (0.18)	3.71	1.99× 10^−11^	2.78× 10^−10^
CMP	0.05 (0.02)	0.13 (0.02)	2.94	1.38 × 10^−8^	8.29 × 10^−8^
2-isopropylmalic acid	0.32 (0.04)	1.30 (0.45)	4.03	7.54× 10^−6^	3.52 × 10^−5^
Glucose	0.04 (0.01)	0.06 (0.01)	1.40	1.41 × 10^−5^	5.37 × 10^−5^
Maltose	1.41 (0.11)	1.60 (0.10)	1.13	2.13 × 10^−3^	5.17 × 10^−3^
UTP	0.04 (0.01)	0.06 (0.01)	1.43	8.79 × 10^−3^	0.02
Glutamate	0.18 (0.04)	0.25 (0.06)	1.41	9.82 × 10^−3^	0.02
Threonine	0.05 (0.01)	0.06 (0.01)	1.22	0.02	0.03
Pantothenic acid	0.13 (0.02)	0.16 (0.02)	1.17	0.03	0.04
Leucine	1.90 (0.25)	0.80 (0.06)	0.42	6.61 × 10^−10^	6.94 × 10^−9^
Lysine	0.59 (0.13)	0.07 (0.02)	0.12	1.53 × 10^−9^	1.29 × 10^−8^
Phenylalanine	0.13 (0.03)	0.02 (0.01)	0.12	1.38 × 10^−8^	8.29 × 10^−8^
dCMP	0.68 (0.08)	0.44 (0.03)	0.65	4.88 × 10^−7^	2.56 × 10^−6^
Succinic acid	2.49 (0.35)	1.67 (0.20)	0.67	1.39 × 10^−5^	5.37 × 10^−5^
AMP	0.70 (0.14)	0.42 (0.05)	0.60	2.92 × 10^−5^	1.02 × 10^−4^
NADP	0.05 (0.01)	0.04 (0.01)	0.74	9.71 × 10^−5^	3.14 × 10^−4^
Formate	0.16 (0.02)	0.12 (0.02)	0.76	2.79 × 10^−4^	8.38 × 10^−4^
N-acetylputrescine	0.16 (0.02)	0.12 (0.02)	0.77	1.11 × 10^−3^	3.10 × 10^−3^
Isoleucine	0.37 (0.12)	0.22 (0.02)	0.61	1.89 × 10^−3^	4.95 × 10^−3^
Acetate	2.50 (0.26)	2.12 (0.18)	0.85	2.22 × 10^−3^	5.17 × 10^−3^
Serine	0.06 (0.01)	0.05 (0.01)	0.87	0.01	0.03

^1^ Abbreviations: GSSG: glutathione disulfide, CMP: cytidine monophosphate, UTP: uridine triphosphate, dCMP: deoxycytidine monophosphate, AMP: adenosine monophosphate, NADP: nicotinamide adenine dinucleotide phosphate, CDP: cytidine diphosphate, NMN: nicotinate mononucleotide, UMP: uridine monophosphate, CTP: cytidine triphosphate, NAD: nicotinamide adenine dinucleotide. ^2^ Values reflect the relative mean area and standard deviation (SD) for the feature assigned to the corresponding metabolite. ^3^ Fold-change provided as *ridA*/wild-type.

**Table 2:** Metabolites below statistical threshold for differential accumulation.

^1^ Compound	^2^ Mean (SD)		^3^ Fold Change	*p* Value	FDR Value
	Wild-Type	*ridA*			
N-acetyl alanine	0.02 (0.01)	0.02 (0.01)	1.24	0.07	0.10
Alanine	0.58 (0.12)	0.68 (0.11)	1.17	0.09	0.12
CDP	0.01 (0.01)	0.02 (0.01)	1.36	0.12	0.15
Uracil	0.06 (0.02)	0.07 (0.03)	1.25	0.22	0.27
Ethanolamine	0.04 (0.01)	0.04 (0.02)	1.15	0.45	0.53
NMN	0.34 (0.08)	0.36 (0.07)	1.05	0.61	0.68
Tyrosine	0.04 (0.02)	0.04 (0.01)	1.06	0.66	0.69
2-oxoglutaric acid	0.08 (0.12)	0.09 (0.16)	1.05	0.95	0.95
Malic acid	0.04 (0.01)	0.03 (0.01)	0.88	0.05	0.07
Putrescine	0.47 (0.21)	0.34 (0.15)	0.72	0.15	0.19
UMP	0.19 (0.04)	0.17 (0.02)	0.93	0.36	0.43
CTP	0.21 (0.04)	0.20 (0.03)	0.95	0.57	0.65
NAD	0.09 (0.04)	0.08 (0.03)	0.92	0.64	0.69
2-aminobutyric acid	1.17 (0.45)	1.14 (0.09)	0.97	0.84	0.86

^1^ Abbreviations: GSSG: glutathione disulfide, CMP: cytidine monophosphate, UTP: uridine triphosphate, dCMP: deoxycytidine monophosphate, AMP: adenosine monophosphate, NADP: nicotinamide adenine dinucleotide phosphate, CDP: cytidine diphosphate, NMN: nicotinate mononucleotide, UMP: uridine monophosphate, CTP: cytidine triphosphate, NAD: nicotinamide adenine dinucleotide. ^2^ Values reflect the relative mean area and standard deviation (SD) for the feature assigned to the corresponding metabolite. ^3^ Fold-change provided as *ridA*/wild-type.

**Table 3:** KEGG pathways differentially regulated between wild-type and *ridA S. enterica.*

Pathway Description	^1^ Relative Abundance	Differential Genes/Total Genes	*p* Value	FDR Value
Thiamine metabolism (stm00730)	*ridA*	8/12	8.60 × 10^−7^	4.80 × 10^−5^
Biotin metabolism (stm00780)	*ridA*	6/14	6.60 × 10^−4^	1.20 × 10^−2^
Biosynthesis of amino acids (stm01230)	*ridA*	18/130	5.50 × 10^−4^	1.50 × 10^−3^
One-carbon pool by folate (stm00670)	*ridA*	5/13	4.40 × 10^−3^	4.90 × 10^−2^
ABC transporters (stm02010)	*ridA*	18/173	1.30 × 10^−2^	8.40 × 10^−2^
Glycine, serine, and threonine metabolism (stm00260)	*ridA*	7/35	1.10 × 10^−2^	8.70 × 10^−2^
Flagellar assembly (stm02040)	WT	31/37	3.00 × 10^−28^	1.40 × 10^−26^
Ribosome (stm03010)	WT	28/78	4.20 × 10^−12^	1.00 × 10^−10^
Bacterial chemotaxis (stm02030)	WT	11/23	3.60 × 10^−6^	5.70 × 10^−5^
Bacterial invasion of epithelial cells (stm05100)	WT	5/9	3.60 × 10^−3^	4.20 × 10^−2^

^1^ Relative abundance denotes the *S. enterica* strain (wild-type or *ridA*) where expression of genes in the corresponding pathway(s) is higher.

## Supplementary Materials

The following are available online at https://www.mdpi.com/2218-1989/10/1/12/s1, Figure S1: Nicotinate mononucleotide identification by sample spiking, Figure S2: Representative 2D J-RES spectrum, Table S1: Identified metabolites and corresponding confidence levels, Table S2: KEGG pathways represented by differentially up-regulated genes in *S. enterica ridA* mutants, Table S3: KEGG pathways represented by differentially down-regulated genes in *S. enterica ridA* mutants.
