# Supplementary material for: Integrated Metabolomics and Transcriptomics Suggest the Global Metabolic Response to 2-Aminoacrylate Stress in Salmonella enterica
Source: Metabolites. 2019 Dec 24;10(1):12. doi: 10.3390/metabo10010012 (PMC7023182; doi:10.3390/metabo10010012)
Supplement: Supplementary file 1 [file metabolites-10-00012-s001.zip › Supplementary Materials/Supplementary Figures.pdf]

Supplementary Material

Integrated metabolomics and transcriptomics suggest the global metabolic response to 2-aminoacrylate stress in *Salmonella enterica*

Andrew J. Borchert[1], Jacquelyn M. Walejko[2], Adrien Le Guennec[3], Dustin C. Ernst[4], Arthur S. Edison[5], and Diana M. Downs[6*]

Department of Microbiology, University of Georgia, Athens, GA 30602

[1] Department of Microbiology, University of Georgia, Athens, GA 30602, USA; Current Address: National Bioenergy Center, National Renewable Energy Laboratory, Golden, CO 90401, USA; aborch@uga.edu
[2] Department of Biochemistry & Molecular Biology, Complex Carbohydrate Research Center, University of Georgia, Athens, GA 30602, USA; Current Address: Duke Molecular Physiology Institute, Duke University, Durham, NC 27701, USA; jacquelyn.walejko@duke.edu
[3] Department of Biochemistry & Molecular Biology, Complex Carbohydrate Research Center, University of Georgia, Athens, GA 30602, USA; Current Address: Centre for Biomolecular Spectroscopy and Randal Division of Cell and Molecular Biophysics, King's College London, New Hunt's House, London, SE1 1UL, United Kingdom; adrien.le_guennec@kcl.ac.uk
[4] Department of Microbiology, University of Georgia, Athens, GA 30602, USA; Current Address: Center for Circadian Biology, University of California, San Diego, CA 92161, USA; dernst@ucsd.edu
[5] Departments of Genetics and Biochemistry & Molecular Biology, Institute of Bioinformatics, Complex Carbohydrate Research Center, University of Georgia, Athens, GA 30602, USA; aedison@uga.edu
[6] Department of Microbiology, University of Georgia, Athens, GA 30602, USA; dmdowns@uga.edu

* Correspondence: dmdowns@uga.edu; Tel.: +1-706-542-9573

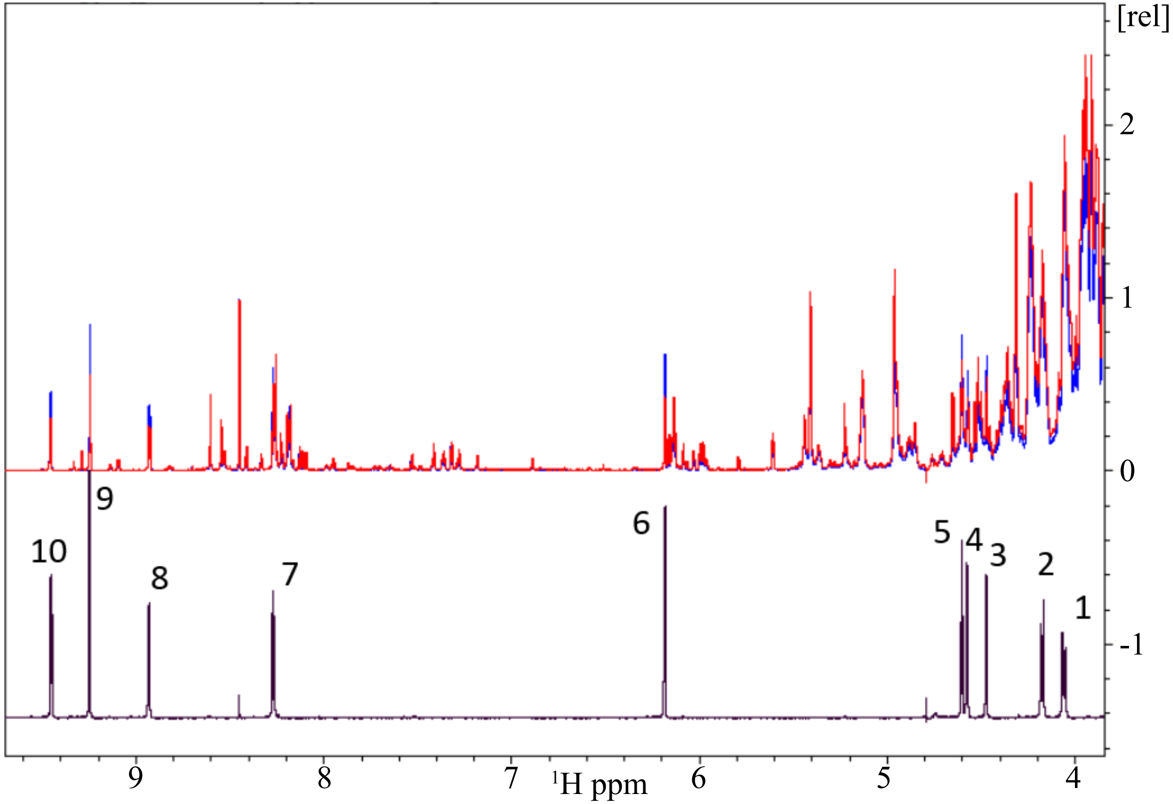

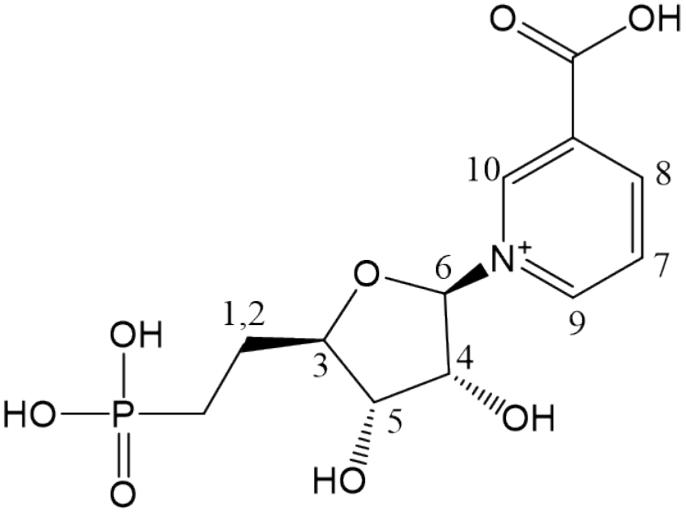

Nicotinate mononucleotide (NMN)

Atom	^{1}H peak	^{13}C peak
1	4.05	65.87
2	4.17	65.87
3	4.47	73.59
4	4.57	90.34
5	4.6	80.36
6	6.18	102.75
7	8.27	131.26
8	8.93	149.55
9	9.25	144.39
10	9.45	143.93

Figure S1. Nicotinate mononucleotide identification by sample spiking. The ^{1}H NMR spectrum was obtained for the pooled metabolite extraction samples with (blue line) and without (red line) the inclusion of 150 µM pure NMN standard. The ^{1}H NMR spectra of NMN alone is provided below (black line). Labeled peaks correspond to labeled atoms on the structural diagram. Exact ^{1}H NMR and ^{13}C peak assignments are listed in the adjacent table.

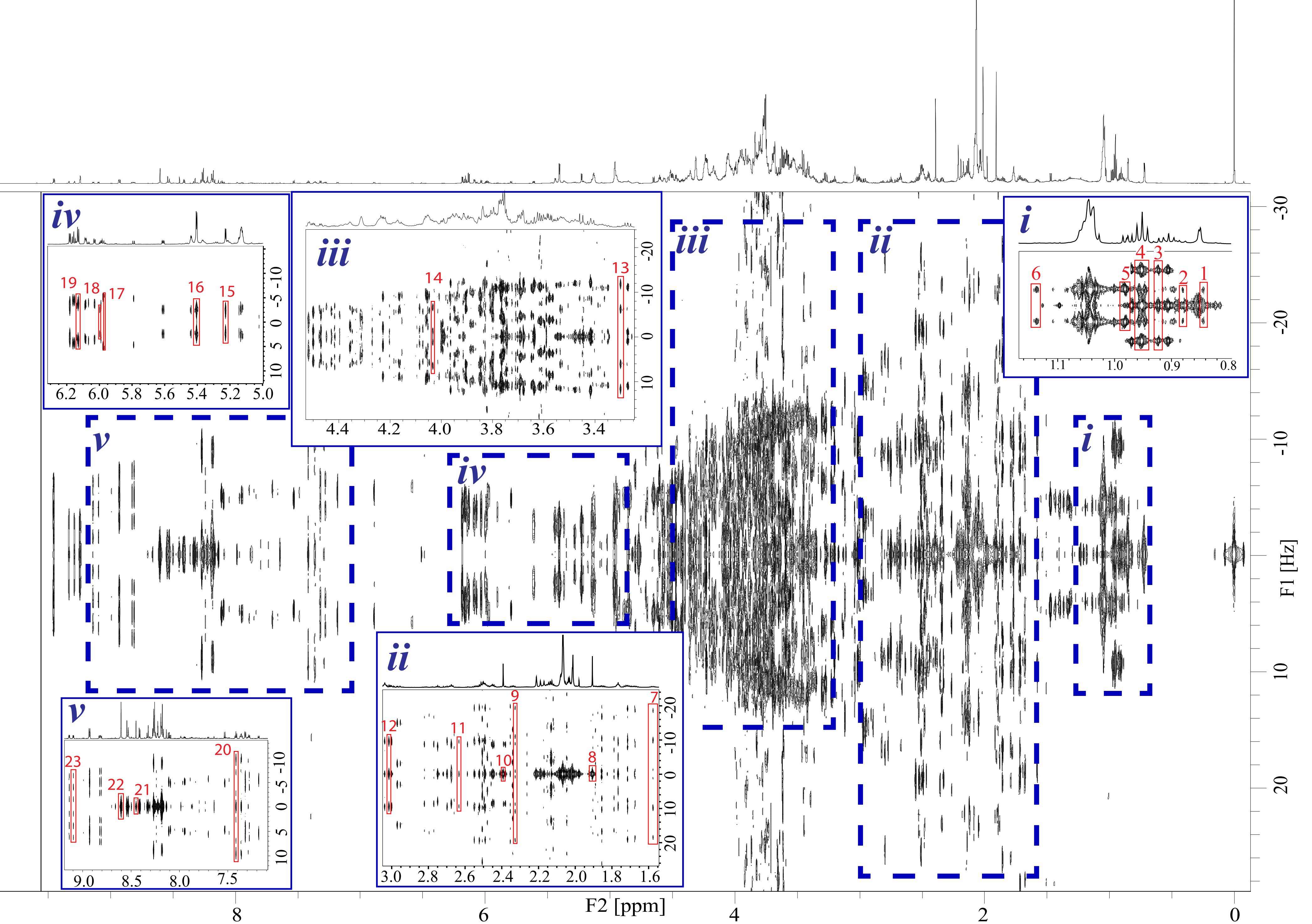

Figure S2. Representative 2D J-RES Spectrum. Features outlined with red boxes were used for quantification: 1) 2-isopropylmalic acid, 2) Pantothenic acid, 3) Ile, 4) Leu, 5) Val, 6) Thr, 7) N-acetylputrescine, 8) Acetate, 9) Glu, 10) Succinate, 11) Met, 12) Lys, 13) GSSG, 14) Ser, 15) Glucose, 16) Maltose, 17) UTP, 18) CMP, 19) dCMP, 20) Phe, 21) Formate, 22) AMP, 23) NAD
